# Chimeric Anti-Staphylococcal Enterotoxin B Antibodies and Lovastatin Act Synergistically to Provide *In Vivo* Protection against Lethal Doses of SEB

**DOI:** 10.1371/journal.pone.0027203

**Published:** 2011-11-15

**Authors:** Mulualem E. Tilahun, Alan Kwan, Kannan Natarajan, Megan Quinn, Ashenafi Y. Tilahun, Chen Xie, David H. Margulies, Barbara A. Osborne, Richard A. Goldsby, Govindarajan Rajagopalan

**Affiliations:** 1 Department of Biology, Amherst College, Amherst, Massachusetts, United States of America; 2 Department of Veterinary and Animal Sciences, University of Massachusetts Amherst, Amherst, Massachusetts, United States of America; 3 Department of Immunology, Mayo Clinic College of Medicine, Mayo Clinic, Rochester, Massachusetts, United States of America; 4 Molecular Biology Section, Laboratory of Immunology, National Institute of Allergy and Infectious Diseases, National Institutes of Health, Bethesda, Maryland, United States of America; The University of Hong Kong, Hong Kong

## Abstract

Staphylococcal enterotoxin B (SEB) is one of a family of toxins secreted by *Staphylococcus aureus* that act as superantigens, activating a large fraction of the T-cell population and inducing production of high levels of inflammatory cytokines that can cause toxic shock syndrome (TSS) and death. Extracellular engagement of the TCR of T-cells and class II MHC of antigen presenting cells by SEB triggers the activation of many intracellular signaling processes. We engineered chimeric antibodies to block the extracellular engagement of cellular receptors by SEB and used a statin to inhibit intracellular signaling. Chimeric human-mouse antibodies directed against different neutralizing epitopes of SEB synergistically inhibited its activation of human T-cells *in vitro*. In the *in vivo* model of lethal toxic shock syndrome (TSS) in HLA-DR3 transgenic mice, two of these antibodies conferred significant partial protection when administered individually, but offered complete protection in a synergistic manner when given together. Similarly, *in vivo,* lovastatin alone conferred only partial protection from TSS similar to single anti-SEB antibodies. However, used in combination with one chimeric neutralizing anti-SEB antibody, lovastatin provided complete protection against lethal TSS in HLA-DR3 transgenic mice. These experiments demonstrate that *in vivo* protection against lethal doses of SEB can be achieved by a statin of proven clinical safety and chimeric human-mouse antibodies, agents now widely used and known to be of low immunogenicity in human hosts.

## Introduction

Staphylococcal enterotoxin B (SEB) is a potent exotoxin secreted by *Staphylococcus aureus* that causes life-threatening toxic shock syndrome (TSS) [1], [2], [3], [4], [5] and food poisoning [6]. Resistant to denaturation, readily produced by recombinant DNA technology and highly toxic (LD_50_ in humans estimated to be nanograms/kg [7], [8]), SEB is classified as a priority B bioterrorism agent. A superantigen, SEB binds to both MHC-II on antigen presenting cells (APCs) and to TCRs incorporating particular Vβ chains on T-cells [2], [3], [4], [9], [10]. The toxin can activate up to 20% of T-cells resulting in the induction of high levels of proinflammatory cytokines, including IL-2, IFN-γ, and TNF-β derived from T_H_1 cells [1], [2], [3], [11], [12], [13] and IL-1 and TNF-α from activated APCs [14], [15], [16]. Its action is initiated by an extracellular phase in which toxin engages the TCR, thereby triggering intracellular signal transduction processes that result in T-cell activation. Several approaches to preventing the formation of MHC- II/SAg/TCR complexes have been explored and include induction of anti-SEB antibodies by immunization with proteosome-SEB toxoid vaccines [17], [18], inactivated recombinant SEB vaccine [19], [20], [21], and synthetic peptides [22], IVIG for passive immunoprophylaxis and immunotherapy [23], [24], [25], [26], peptide antagonists [12], [27], [28], and synthetic chimerically linked mimics of SEB-binding regions of class II and TCR [29], [30], [31]. Engineered mimics of TCR Vβ [32] that block SEB activation *in vitro* and show promising results when tested *in vivo* in a rabbit model have been reported [32]. However, these mimics were reported to have short half-lives (325 minutes in rabbits) and their test in human MHC-II transgenics, a robust animal model that mimics human TSS [33], [34], [35], [36], [37], [38] has not yet been reported. Despite these efforts, at present there is no curative treatment for SEB-induced TSS, no practical prophylaxis and no antidote for intoxication following accidental or malicious exposure. The mortality rate varies from 4 to 22% and clinical treatment is currently focused on supportive measures, targeted antibiotic therapy, and adjunctive immunomodulatory therapy [39].

We recently generated high affinity human-mouse chimeric monoclonal antibodies (MAbs) against SEB. We have shown that these antibodies are capable of neutralizing SEB *in vitro*, and attenuate SEB-induced immune activation *in vitro* [40]. Subsequent to our report, there have been studies describing the generation of additional anti-SEB antibodies [41], [42]. More recently, Varshney et al., described the generation and characterization of murine monoclonal antibodies with neutralizing and protective abilities against SEB-induced lethal shock (SEBILS) [43]. In the current study we establish the neutralizing potential of our human-mouse chimeric antibodies *in vivo* and also show that our chimeric anti-SEB antibodies are able to protect from lethal SEB-induced TSS in a more robust HLA-DR3 transgenic mice model. In addition, we examined the possibility that an intracellular inhibitor of T-cell activation and cytokine signaling would complement the inhibitory effect of extracellularly acting anti-SEB antibody. As an intracellular inhibitor of SEB-induced signal transduction processes, we used lovastatin, and found this statin inhibited T-cell activation just as the structurally similar simvastatin has been shown to do [44]. Lovastatin (Mevacor™) is widely used in clinical practice and is known to have low toxicity in humans [45]. In addition to their well known role in reduction of cholesterol levels, statins are known also to have anti-inflammatory and immunomodulatory properties [44], [46]. Simvastatin is reported to inhibit SEB-mediated T-cell activation in human peripheral blood [44], and atorvastatin enhances T-cell differentiation from T_H_1 to T_H_2 [47]. Statins also inhibit cytokine-mediated signaling pathways [48].

## Results

### Chimeric Anti-SEB Antibodies Protect Mice from SEB-induced TSS More Effectively in Combination than Alone

In our previous report, we identified a pair of high affinity, non-crossreacting, and SEB-neutralizing mouse MAbs and then converted these antibodies into the mouse-human chimeric antibodies, Ch 82 M and Ch 63 [40]. When we tested the SEB-neutralization efficiency of these chimeric antibodies *in vitro* in splenocyte cultures derived from HLA-DR3 transgenic nice, a more demanding and humanlike model system [35], [36], [37], [49] as well as in human PBMCs, a combination of Ch 82 M and Ch 63 produced a greater neutralization of SEB than equivalent amounts of either 82 M or Ch 63 acting alone [40].

SEB binds human MHC-II more strongly than mouse [11], [50]. Rajagopalan [36], [37] and others [35], [49] have shown that HLA-DR3 transgenic mice, engineered to express human instead of mouse class II MHC, provide a more stringent model system for the exploration and development of anti-SEB strategies than conventional mice. Moreover, unlike the conventional animal models, HLA-DR3 transgenic mice do not require sensitizing agents to induce TSS with SEB. Challenging naïve HLA-DR3 transgenic mice with 50 µg of SEB causes them to undergo a rapid and sharp drop in body temperature (hypothermia), produce an excess amount of inflammatory cytokines and die within 7 days. The immunopathogenesis of TSS in HLA-DR3 transgenic mice more closely mimics the human disease [33], [35]. Therefore, we performed *in vivo* studies in HLA-DR3 transgenic mice to examine the efficacy of these chimeric antibodies in protecting mice from SEB-induced excessive systemic cytokine production, TSS symptoms and lethality.

**Figure 1:**
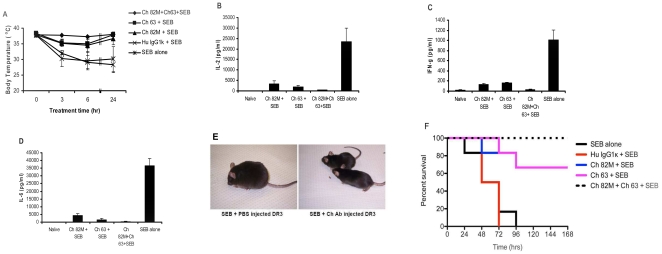
Chimeric anti-SEB 82 M and 63 synergistically protect HLA-DR3 mice from the toxic effects of SEB. (**A**) Protection against SEB-induced hypothermia. Age-matched HLA-DR3 transgenic mice were injected with the following: Ch 82 M+Ch 63+SEB (♦), 500 µg chimeric 82 M+500 µg chimeric 63+50 µg SEB; Ch 63+SEB (▪), 1 mg chimeric 63+50 µg SEB; Ch 82 M+SEB (▴),1 mg chimeric 82 M+50 µg SEB; Hu IgG1κ+SEB (×), 1 mg human IgG1κ+50 µg SEB; SEB alone (*); 50 µg SEB in PBS. Rectal temperatures were recorded at the indicated time points. Error bars are the means ± s.d. for each group, and the data shown are representative of two or more independent experiments. **(B**–**D)** Inhibition of SEB-induced cytokine production. Serum levels of interleukin-2 (**B**), interferon-γ (**C**), and interleukin-6 (**D**) were determined at 6 hours post-SEB injection in groups of age-matched HLA-DR3 transgenic mice treated with the following: naïve, PBS only; Ch 82 M+SEB, 1 mg Ch 82 M+50 µg SEB; Ch 63+SEB, 1 mg Ch 63+50 µg SEB; Ch 82 M+Ch 63+SEB, 500 µg Ch 82 M+500 µg Ch 63+50 µg SEB; SEB alone, 50 µg of SEB in PBS. Differences between the combination of anti-SEBs and either antibody used alone were significant (P<0.05) in inhibiting SEB-induced cytokine production. Each bar represents the means ± s.d. for each group, and the data shown are representative of two or more independent experiments. (**E**) Appearance of mice protected with anti-SEB and unprotected mice 6 hours after a 50 µg dose of SEB. The mouse on the left received no protective antibody, suffered hyperthermia, shivered and displayed hunched posture and a rough coat. The mice on the right, which were treated with 50 µg SEB+500 µg Ch 82 M+500 µg Ch 63 appeared normal, were sleek of coat and animated. (**F**) Protection against SEB-mediated death. Survival was monitored within groups of age-matched HLA-DR3 transgenic mice receiving the following: SEB alone, 50 µg SEB in PBS; Hu IgG1κ+SEB, 1 mg human IgG1κ+50 µg SEB; Ch 82 M+SEB, 1 mg Ch 82 M+50 µg SEB; Ch 63+SEB, 1 mg Ch 63+50 µg SEB; Ch 82 M+Ch 63+SEB, 500 µg Ch 82 M+500 µg Ch 63+50 µg SEB. Combination of Ch 82 M and Ch 63 provided statistically significant protection (P<0.001) against SEB-induced death compared with untreated controls. The data shown are representative of two or more independent experiments.

We challenged HLA-DR3 transgenic mice with SEB alone, or SEB along with chimeric anti-SEB or human IgG1κ isotype control antibody. We monitored the body temperature of the mice for 24 hours by recording rectal temperature at the indicated time points. While either Ch 82 M or Ch 63 significantly decreased (P<0.05) hypothermia, mice treated with an equivalent amount of an equimolar mixture of Ch 82 M plus Ch 63, experienced no drop in temperature 6 hours after SEB administration indicating the synergistic effect of the two antibodies (P<0.01; **Fig. 1A**). On the other hand, the body temperatures of SEB challenged mice treated with PBS or human IgG1κ isotype control fell abruptly in the first 6 hours and remained below normal for 24 hrs (**Fig. 1A**).

We next investigated the effect of our chimeric antibodies on SEB-induced systemic cytokine induction. Since interleukin- 2 (IL-2), and particularly interferon-γ (IFN-γ), play major roles in SEB-induced toxic shock syndrome in HLA-DR3 transgenic mice [33], these cytokines were chosen. Mice injected with SEB alone, SEB+Chimeric anti-SEB MAbs, or untreated control were bled 6 hours after injection and serum levels of IL-2, IFN-γ and interleukin- 6 (IL-6) were determined by Bioplex ELISA. Naïve HLA-DR3 transgenic mice had undetectable levels of IL-2, IFN-γ and IL-6, whereas mice challenged with SEB alone resulted in a significant elevation of these cytokines a few hours after injection of SEB, indicative of the acute course of SEB-induced TSS. When separately administered, however, either Ch 82 M or Ch 63 alone significantly reduced SEB-induced production of IL-2, IFN-γ and IL-6 (P<0.01). However, an equivalent amount of the combination of Ch 82 M+Ch 63 provided a greater reduction of cytokine secretion (P<0.05) compared with either antibody alone, reducing cytokines levels that did not significantly differ (*P*>0.05) from those of mice that received no SEB (**Fig. 1B**–**D**). Activity of HLA-DR3 transgenic mice was monitored after injection of 50 µg of SEB in PBS, or with chimeric anti-SEB antibodies. After 6 hours, mice treated with SEB alone were hypothermic, shivered, had hunched posture, were inactive or unable to move around and had rough coats. On the other hand, mice treated with 50 µg SEB+500 µg Ch 82 M+500 µg Ch 63 appeared normal, were sleek of coat, energetic and mobile (**Fig. 1E**).

We then monitored mice treated with SEB alone, or SEB along with chimeric or isotype control antibodies for a period of 7 days to record the lethal effect of SEB. Notably, 66% of the mice receiving a fatal dose of SEB survived if treated with either Ch 82 M or Ch 63 and all mice treated with equivalent amounts of the combination of Ch 82 M plus Ch 63 survived (100%), whereas mice that were injected with SEB alone or SEB plus nonspecific control human IgG1κ, all succumbed to SEB toxicity (**Fig. 1F** **)**. Treatment with a combination of Ch 82 M and Ch 63 provided statistically significant (P<0.001) protection against SEB-induced death.

### Effect of Lovastatin on Reducing SEB-induced T-cell Proliferation *In Vitro* and its Synergy with Chimeric Anti-SEB Antibody

**Figure 2:**
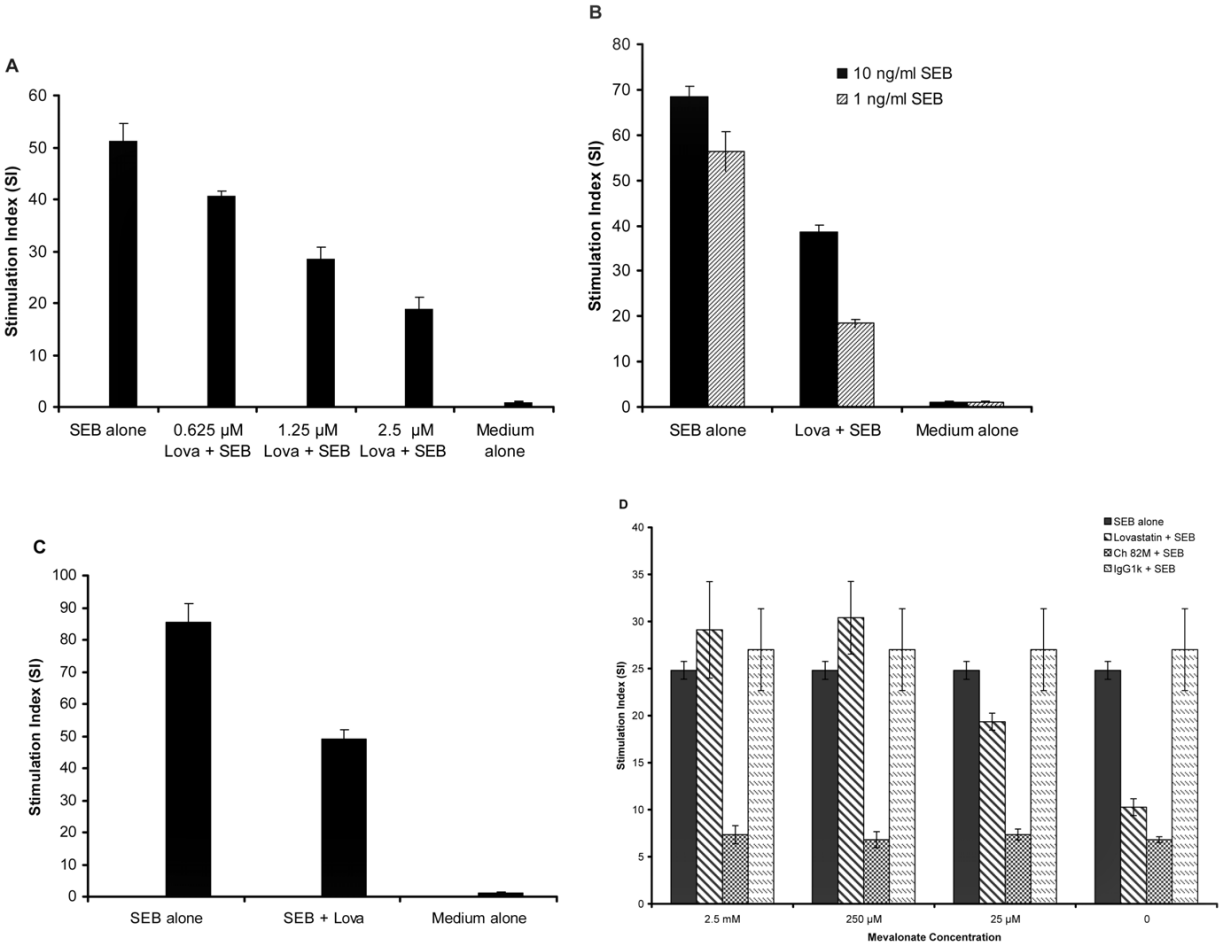
Neutralization of SEB-induced T-cell proliferation by lovastatin. (**A**) ***In vitro*** neutralization in BALB/c splenocytes. BALB/c splenocytes (5×105 cells/well) were incubated at 37°C for 48 hours in the presence of medium alone, SEB alone (100 ng/ml), SEB+different concentrations of lovastatin (0.625 µM, 1.25 µM, and 2.5 µM) as indicated. Cells were then pulsed with 3H-thymidine (1 µci/well), incubated for an additional 18 hours, harvested and cell proliferation determined by measuring 3H-thymidine incorporation. Each bar represents the means ± s.d. of triplicate measurements, and the data shown are representative of two or more independent experiments. Neutralization of SEB-induced T-cell proliferation by lovastatin were significant (P<0.01) compared with results obtained with SEB alone. (**B**) SEB neutralization in HLA-DR3 transgenic mice splenocytes. HLA-DR3 splenocytes (5×105 cells/well) were incubated at 37°C for 48 hours in the presence of medium alone, with various concentration of SEB alone (10 ng/ml and 1 ng/ml), or SEB plus lovastatin (2.5 µM). Cells were then pulsed with 3H-thymidine (1 µci/well), incubated for an additional 18 hours, harvested and cell proliferation determined by measuring 3H-thymidine incorporation. Each bar represents the means ± s.d. of triplicate measurements, and the data shown are representative of two or more independent experiments. Neutralization of SEB-induced T-cell proliferation by lovastatin were significant (***P***<0.01) compared to cells treated with SEB alone. (**C**) SEB neutralization in human PBMC. Human PBMC (5×105 cells/well) were incubated at 37°C for 48 hours in the presence of medium alone, SEB alone (1 ng/ml), or SEB plus lovastatin (2.5 µM). Cells were then pulsed with 3H-thymidine (1 µci/well), incubated for an additional 18 hours, harvested and cell proliferation determined by measuring 3H-thymidine incorporation. Each bar represents the means ± s.d. of triplicate measurements, and the data shown are representative of two or more independent experiments. Neutralization of SEB-induced T-cell proliferation by lovastatin were significant (***P***<0.01) compared to cells treated with SEB alone. (**D**) Mevalonate mediated reversal of lovastatin's inhibition of SEB in BALB/c splenocytes. Bar groups are organized by the amount of mevalonate added to each well. BALB/c splenocytes (5×105 cells/well) were incubated at 37°C for 48 hours with various concentration of mevalonate (25 mM, 250 µM, 25 µM or 0), and in the presence of SEB alone (100 ng/ml SEB); lovastatin+SEB (2.5 µM lovastatin+100 ng/ml SEB); Ch 82 M+SEB (10 µg/ml chimeric anti-SEB 82 M+100 ng/ml SEB); IgG1κ+SEB (10 µg/ml human IgG1κ isotype control+100 ng/ml SEB). Cells were then pulsed with 3H-thymidine (1 µci/well), incubated for an additional 18 hours, harvested and cell proliferation determined by measuring 3H-thymidine incorporation. Each bar represents the means ± s.d. of triplicate measurements, and the data shown are representative of two or more independent experiments. Inhibition of SEB by lovastatin was not significant (P>0.05) at 25 µM mevalonate.

The ability of lovastatin to inhibit SEB-induced T-cell proliferation was examined *in vitro* in BALB/c and HLA-DR3 transgenic mice splenocytes and human PBMC cultures. The results of the experiments revealed that lovastatin neutralized SEB-induced T-cell proliferation significantly (P<0.01) compared to cells treated with SEB alone (**Fig. 2A**–**C**). To confirm the likelihood that the inhibitory effect of lovastatin is due to its effect on HMG-CoA reductase, the enzyme targeted by statins, we added mevalonate, the product of HMG-CoA reductase, to determine if it reversed the effects of lovastatin. The result demonstrates the ability of mevalonate to counter the protective effect of lovastatin and indicates that the inhibitory effect of this statin on SEB-mediated T-cell activation strongly impacts the mevalonate pathway (**Fig. 2D**).

**Figure 3:**
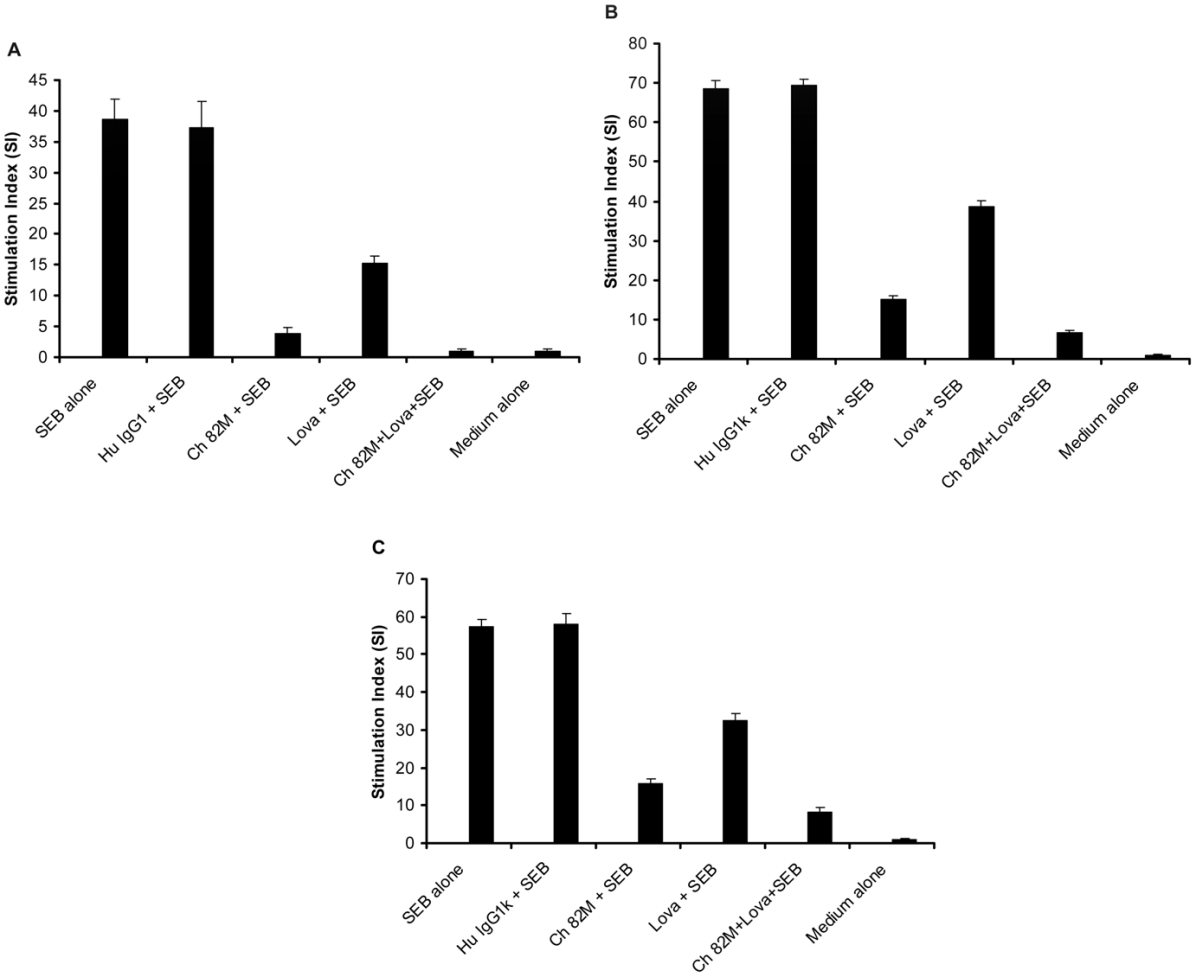
Synergistic neutralization /inhibition of SEB-mediated T-cell activation by a combination of anti-SEB antibody and lovastatin. (**A**) Anti-SEB Ch 82 M and lovastatin inhibit SEB action in BALB/c splenocytes. BALB/c splenocytes were cultured as outlined in materials and methods with the following additives: Medium alone, SEB alone, 100 ng/ml SEB; Hu IgG1κ+SEB, 10 µg/ml Human IgG1κ+100 ng/ml of SEB; Ch 82 M+SEB, 10 µg/ml chimeric anti-SEB 82 M+100 ng/ml SEB; Lova+SEB, 2.5 µM lovastatin+100 ng/ml SEB; Ch 82 M+Lova+SEB, 10 µg /ml chimeric anti-SEB 82 M+2.5 µM lovastatin+100 ng/ml SEB. Each bar represents the means ± s.d. of triplicate measurements, and the data shown are representative of two or more independent experiments. The combination of Ch 82 M+lovastatin was significantly more inhibitory than either agent alone (P<0.01). (**B**) Anti-SEB Ch 82 M and lovastatin inhibit SEB action in HLA-DR3 transgenic mice splenocytes. HLA-DR3 transgenic mice splenocytes were cultured as outlined in materials and methods with the following additives: Medium alone, SEB alone, 10 ng/ml SEB; HuIgG1κ+SEB, 10 µg/ml Human IgG1κ+10 ng/ml of SEB; Ch 82 M+SEB, 10 µg/ml chimeric anti-SEB 82 M+10 ng/ml SEB; Lova+SEB, 2.5 µM lovastatin+10 ng/ml SEB; Ch 82 M+Lova+SEB, 10 µg/ml chimeric anti-SEB 82 M+2.5 µM lovastatin+10 ng/ml SEB. Each bar represents the means ± s.d. of triplicate measurements, and the data shown are representative of two or more independent experiments. The combination of Ch 82 M and lovastatin was significantly more inhibitory than either agent alone (P<0.01). (**C**) Anti-SEB Ch 82 M and lovastatin inhibit SEB action in human PBMCs. PBMCs were cultured as outlined in materials and methods with the following additives: Medium alone, SEB alone, 1 ng/ml SEB; HuIgG1κ+SEB, 10 µg/ml Human IgG1κ+1 ng/ml of SEB; Ch 82 M+SEB, 10 µg/ml chimeric anti-SEB 82 M+1 ng/ml SEB; Lova+SEB, 2.5 µM lovastatin+1 ng/ml SEB; Ch 82 M+Lova+SEB, 10 µg/ml chimeric anti-SEB 82 M+2.5 µM lovastatin+1 ng/ml SEB. Each bar represents the means ± s.d. of triplicate measurements, and the data shown are representative of two or more independent experiments. The combination of Ch 82 M and lovastatin was significantly more inhibitory than either agent alone (P<0.01).

Further *in vitro* experiments revealed that combinations of Ch 82 M and lovastatin caused a greater reduction of SEB-induced T-cell proliferation (P<0.01) than either treatment alone in mouse splenocyte cultures (**Fig. 3A**), in HLA-DR3 transgenic mice splenocyte cultures (**Fig. 3B**), and in human PBMCs (**Fig. 3C**).

### Combinations of Chimeric Anti-SEB Antibody and Lovastatin Protect HLA-DR3 Transgenic Mice from SEB-induced TSS Better than Either Agent Alone

**Figure 4:**
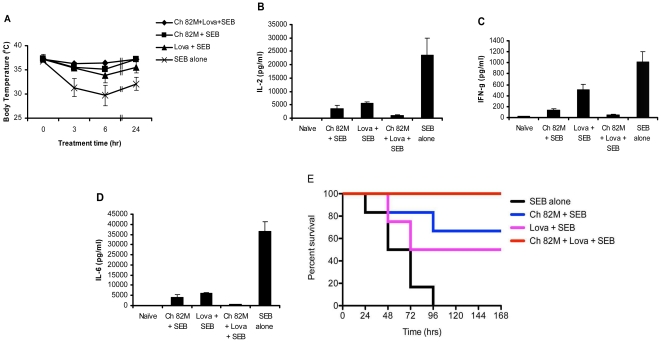
Chimeric anti-SEB 82M and lovastain provide *in vivo* protection against SEB toxicity in HLA-DR3 transgenic mice. (**A**) Inhibition of SEB-mediated hypothermic effect. Age-matched HLA-DR3 transgenic mice were injected with the following: Ch 82 M+Lova+SEB (♦), 1 mg chimeric 82 M+1 mg lovastatin+50 µg SEB; Ch 82 M+SEB (▪), 1 mg chimeric 82 M+50 µg SEB; Lova+SEB (▴), 1 mg lovastatin +50 µg SEB; SEB alone (×), 50 µg SEB in PBS. Rectal temperatures were recorded at the indicated time points. Error bars are the means ± s.d. for each group, and the data shown are representative of two or more independent experiments. (**B**–**D**) Inhibition of SEB-induced cytokine production. Serum levels of interleukin-2 (**B**), interferon-γ (**C**) and interleukin-6 (**D**) were determined at 6 hours post-SEB injection in groups of age-matched HLA-DR3 transgenic mice treated with the following: naïve, PBS only; Ch 82 M + SEB, 1 mg Ch 82 M+50 µg SEB; Lova + SEB, 1 mg lovastatin +50 µg SEB; Ch 82 M + Lova + SEB, 1 mg Ch 82 M+1 mg lovastatin+50 µg SEB; SEB alone, 50 µg SEB in PBS. Differences between the combination of Ch 82 M and lovastatin and either drug or antibody alone were significant (P<0.05) in inhibiting SEB-induced cytokine production. Each bar represents the means ± s.d. for each group, and the data shown are representative of two or more independent experiments. (**E**) Protection against SEB-mediated death. Survival was monitored within groups of age-matched HLA-DR3 transgenic mice receiving the following: SEB alone, 50 µg SEB in PBS; Ch 82+SEB, 1 mg of Ch 82 M+50 µg SEB; Lova+SEB, 1 mg of lovastatin+50 µg SEB; Ch 82 M+Lova + SEB, 1 mg Ch 82 M+1 mg lovastatin+50 µg SEB. Survival was monitored for 7 days. Combination of Ch 82M and lovastatin provided statistically significant protection (P<0.01) against SEB-induced death compared with mice treated with SEB alone. The data shown are representative of two or more independent experiments.

Since our *in vitro* experiments demonstrated efficient neutralization of SEB-induced T-cell activation by a combination of chimeric anti-SEB, (an extracellular inhibitor), and lovastatin (an intracellular inhibitor), we examined the possibility of translating our *in vitro* SEB neutralization findings into *in vivo* protection against SEB-mediated illness and mortality. Similar to the above experiment using a combination of two chimeric anti-SEB antibodies, the possibility of cooperative or synergistic inhibition of SEB action *in vivo* by anti-SEB antibody and lovastatin was investigated in the HLA- DR3 transgenic mice TSS model. We evaluated the effect of these agents on SEB-mediated hypothermia, serum cytokine (IL-2, IFN-γ, and IL-6) levels and mortality. **Figure 4A** shows that 6 hours after SEB administration, the combination of Ch 82 M and lovastatin was superior to Ch 82 M or lovastatin alone (P<0.05) in ameliorating hypothermia. Although Ch 82 M or lovastatin inhibited IL-2 accumulation as well as that of the inflammatory cytokines IFN-γ and IL-6, combinations of Ch 82 and lovastatin were more effective than either agent alone (P<0.01) in inhibiting SEB-induced cytokine production (**Fig. 4B-D**). Most importantly, combination of Ch 82 M and lovastatin conferred full protection against SEB mediated death, where as Ch 82 M conferred 66% protection, lovastatin conferred 50% protection and all mice that did not receive treatment were victims of SEB lethality (**Fig. 4E**). Treatment with a combination of Ch 82 M and lovastatin conferred statistically significant (P<0.01) protection against SEB-induced death.

## Discussion

SEB-mediated diseases are the result of the activation of a large subset of the T-cell population. When the SEB crosslinks the MHC class II of the APCs to the Vβ TCR of T-cells to form MHC II-SEB-TCR complexes, it results in the activation of T-cells and induction of massive systemic release of inflammatory cytokines. Hence, blockade of SEB binding to either receptor prevents formation of the MHC II-SEB-TCR complex and inhibits the superantigenic action of SEB. Therefore, therapeutic approaches targeting disruption of MHC II-SEB-TCR complex formation at an early stage of the pathogenic process could prevent, minimize, or ameliorate the severity and incidence of SAg-caused diseases. In this report, we used anti-SEB antibodies reported previously [40] to prevent binding of SEB to its receptors and showed that they were able to efficiently neutralize SEB-induced proinflammatory cytokine production *in vivo,* and prevent SEB-mediated disease and death in an HLA-DR3 transgenic animal model. Our findings demonstrated the synergistic action of the two antibodies in neutralizing SEB-mediated TSS *in vivo* in a readily available and robust animal model. These findings encourage the further evaluation of these approaches for the treatment and prevention of TSS in humans.

Given the rapidity with which SEB causes T-cell activation and elicits cytokine/chemokine production *in vivo*, relying only on neutralization of SEB does not take advantage of simultaneously inhibiting independent processes essential for SEB-mediated T-cell activation. We tested the hypothesis that a multipronged attack aimed at disrupting the formation of MHC II-SEB-TCR complex, an approach inhibiting the signaling events elicited in T-cells (and APC) following this interaction as well as inhibiting the downstream signaling events in target tissues/cells in response to the systemic cytokine/chemokine storm elicited by SEB, would be more effective. Given the known immunomodulatory functions of statins and their established ability to inhibit T-cell as well as cytokine/chemokine signaling, we evaluated lovastatin in this study. Although identifying all of the molecular mechanisms by which lovasatin inhibits SEB-induced T-cell activation and the cytokine/chemokine signaling is difficult and would require further investigation, some of the possible explanations can be deduced from work reported in the literature.

Lovastatin (Mevacor™) is a widely prescribed drug for treatment of hypercholesterolemia. Recent studies indicate that statins also have anti-inflammatory and immunomodulatory functions [44], [46]. Affecting intracellular processes, statins decrease IFN-γ-induced expression of MHC class II on APCs [51], blocks the interaction between LFA-1 on APC and ICAM-1 expressed on T- cells [52], and decreases HLA-DR and CD38 expression on T-cells [44]. MHC class II expression on APCs is important for SEB to bind as its primary target receptor and to crosslink it to T-cells expressing Vβ TCR to form a complex and induce T-cell activation. A statin-mediated decrease in the expression of MHC class II would ultimately decrease the number of MHC II –SEB-TCR complexes formed and lower the number of T-cells activated. Similarly, LFA-1, ICAM-1 interaction is required for SEB-induced TSS [53]. The effect of another statin, simvastatin in inhibiting SEB-mediated T-cell activation in cultures of human PBMC, may be related to inhibition of LFA-1 [44]. Therefore, given the significance of APC-T-cell interaction pathways in activation by SEB, lovastatin could antagonize the effects of SEB by inhibiting this interaction. In addition, statins are known to promote a T_H_1 toT_H_2 polarization [47]. Such a mechanism could ameliorate the severity of TSS since SEB-induced pathology is thought to be caused by the proinflammatory cytokine storm derived mainly from T_H_1 cells [1], [2], [3], [11], [12], [13].

In addition to its effect on T-cells, statins could mediate its anti-inflammatory effect on the innate immune system by inhibiting pro-inflammatory signals from many cytokines and chemokines that are produced during TSS [48]. Taken together, all of these effects suggest that statins have the potential to inhibit TSS by a variety of mechanisms. Consistent with our laboratory findings, retrospective human studies have shown that patients who were previously taking statins had better outcomes following sepsis or trauma [54].

Given the widespread use of chimeric antibodies in human patients to treat a variety of clinical conditions and the fact that lovastatin has been in clinical use in humans for many years, our suggestion of a therapeutic approach incorporating these agents would be expected to be well-tolerated in clinical settings and, may be suitable for widespread administration to populations under threat of malevolent use of SEB. Looking beyond biodefense, SEB is known to be produced during many *Staphylococcus aureus* infections *in vivo*. Given that *S. aureus* is the leading cause of infections in hospitalized patients [55], exploration of the clinical use of our antibodies either alone or in combination with lovasatin or other immunomodulatory agents, is worthy of consideration. Therefore, incorporating a well tolerated inhibitor of the intracellular processes necessary for the action of superantigens, such as lovastatin, encourages investigation of its use in combination therapies for the prophylaxis and therapy of disease caused by clinically significant representatives of these bacterial toxins. Also, the derivation and chimerization of neutralizing antibodies against other staphylococcal (and streptococcal) superantigen toxins is quite feasible. Once produced, they can be added to those reported here to provide a polyclonal mixture of anti-toxin antibodies, a combination broadly protective against a variety of these superantigens and likely to be synergistically active with agents such as lovastatin.

## Materials and Methods

### Ethics Statement

All animal experimentation was carried out in strict accordance with the recommendations in the Guide for the Care and Use of Laboratory Animals of the National Institutes of Health. The protocols were approved by the Institutional Animal Care and Use Committee of Mayo Clinic (Approval number: A26408, A29507 and A31810). The AAALAC Accreditation Number is 000717 and the OLAW Assurance Number is A3291-01. All efforts were made to minimize suffering.

### Mice

BALB/c (*H-2^d^*) or HLA-DRB1*0301 mice were sources of splenocytes for some *in vitro* neutralization studies. HLA-DR3 transgenic mice expressing functional HLA-DRA1*0101 and HLA-DRB1*0301 transgenes on a completely mouse MHC-II-deficient background (AE^0^) were generated as described elsewhere [36], [56], [57].

### SEB

Endotoxin-reduced SEB was purchased from Toxin Technology (Sarasota, FL), and laboratory stocks were always less than the federally mandated limit of 5 mg. The manufacturer has certified that the SEB batch purchased has>95% purity on the basis of gel analysis. In house SDS-PAGE confirmed the purity of SEB.

### *In Vitro* SEB Neutralization

Splenic mononuclear cells from BALB/c or HLA- DR3 transgenic mice were isolated by Ficoll-Paque Plus (GE Healthcare, Uppsala, Sweden) gradient centrifugation and washed 2X with PBS and resuspended in growth medium (RDGS) containing 45% DMEM, 45% RPMI 1640, 10% fetal bovine serum (FBS), 55 µM 2ME (all from Invitrogen, Carlsbad, CA), and 20 µg/ml gentamicin (Sigma-Aldrich, St. Louis, MO). Human PBMCs were purchased from Stem Cell Technologies (Vancouver, Canada). Cell suspensions (5×10^6^ cells/ml) were distributed in 100 µl aliquots to the wells of 96-well flat-bottom plates (5×10^5^ cells/well) containing 50 µl of various concentrations of SEB and 50 µl of the indicated concentration of anti-SEB antibody and/or the indicated concentration of lovastatin. Each condition was run in triplicate. The cells were incubated (at 37°C in 7.5% CO_2_) for 48 hours and then pulsed with 1 µCi ^3^H- thymidine (Amersham/GE Healthcare) per well, incubated an additional 18 hours and then harvested onto glass fiber filter strips with a PhD cell harvester (Brandel, Gaithersburg, MD). The incorporated radioactivity was measured by liquid scintillation counting and stimulation indices (SI) were calculated as;
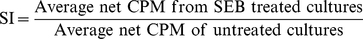



### *In Vivo* TSS Study

Age-matched HLA-DR3 transgenic mice were challenged with 50 µg SEB in PBS by the intraperitoneal(i.p.) route immediately following injection of the indicated treatments of PBS, isotype control (human IgG1κ), chimeric anti-SEB antibodies (Ch 63 and Ch 82 M), and/or lovastatin. Body temperatures and survival were determined at the indicated time intervals. Blood samples collected at 6 hrs after the indicated treatment were tested according to the manufacturers' instructions in a Bio-plex cytokine ELISA (Bio-Rad Laboratories, Hercules, CA) to determine the serum levels of IL-2, IFN-γ and IL-6.

### Statistical Methods

Mean values of stimulation index (SI), body temperature, and cytokine levels were compared using Student's *t* test with P values<0.05 considered significant. Survival curves and their statistical analysis were generated using the GraphPad Prism software (version 5.0d; San Diego, CA).
